# NFAT4-dependent miR-324-5p regulates mitochondrial morphology and cardiomyocyte cell death by targeting Mtfr1

**DOI:** 10.1038/cddis.2015.348

**Published:** 2015-12-03

**Authors:** K Wang, D-l Zhang, B Long, T An, J Zhang, L-Y Zhou, C-Y Liu, P-F Li

**Affiliations:** 1 Center for Developmental Cardiology, Institute for Translational Medicine, College of Medicine, Qingdao University, Qingdao, China; 2 Department of Cardiology, Shanghai Chest Hospital, Shanghai Jiaotong University, Shanghai, China; 3 Laboratory of Molecular Medicine, Central Research Laboratory, Peking Union Medical College Hospital, Chinese Academy of Medical Sciences and Peking Union Medical College, Beijing, China; 4 State Key Laboratory of Cardiovascular Disease, Heart Failure Center, Fuwai Hospital, National Center for Cardiovascular Diseases, Chinese Academy of Medical Sciences, Peking Union Medical College, Beijing, China

## Abstract

Emerging evidence suggest that the abnormal mitochondrial fission participates in pathogenesis of cardiac diseases, including myocardial infarction and heart failure. However, the molecular components regulating mitochondrial network in heart remain largely unidentified. Here we report that NFAT4, miR-324-5p and mitochondrial fission regulator 1 (Mtfr1) function in one signaling axis that regulates mitochondrial morphology and cardiomyocyte cell death. Knocking down Mtfr1 suppresses mitochondrial fission, apoptosis and myocardial infarction. Mtfr1 is a direct target of miR-324-5p, and miR-324-5p attenuates mitochondrial fission, cardiomyocyte apoptosis and myocardial infarction by suppressing Mtfr1 translation. Finally, we show that transcription factor NFAT4 inhibits miR-324-5p expression. Knockdown of NFAT4 suppresses mitochondrial fission and protects cardiomyocyte from apoptosis and myocardial infarction. Our study defines the NFAT4/ miR-324-5p/Mtfr1 axis, which participates in the regulation of mitochondrial fission and cardiomyocyte apoptosis, and suggests potential new treatment avenues for cardiac diseases.

Cardiac disease remains the leading cause of death for people worldwide. Heart pumps oxygen and nutrient-rich blood throughout the body to sustain life and has an extensive requirement for energy production. Many studies unveil that cardiomyocytes possess large amounts of mitochondria. Mitochondria ensure the continuous aerobic respiration and production of ATP for cardiac function. Mitochondria forms a complex network by fission and fusion,^1^ and mitochondrial network is associated with various cellular process, such as embryo development, cell differentiation, apoptosis and necrosis.^2, 3, 4, 5^ Mitochondrial network dynamics, particularly, mitochondrial fission and fusion, participate in maintaining mitochondrial function.^6^ The disruption of mitochondrial fission and fusion is related to several human diseases, including neurodegeneration, cardiovascular diseases and cancer.^7, 8, 9^ Accumulating lines of evidence indicate that cardiac diseases are associated with mitochondrial dysfunction.^8, 10, 11^ Mitochondrial fission leads to the formation of small round mitochondria and promotes cell apoptosis, whereas fusion results in mitochondria elongation and have a protective role in cardiomyocytes maintenance.^8, 11^ These findings strongly suggest that mitochondrial dynamics is important for cardiac function. Thus, unveiling the mechanisms of mitochondrial network regulation will provide a novel therapeutic strategy for cardiovascular diseases and heart failure.

Mitochondrial fission regulator 1 (Mtfr1) is a mitochondrial protein containing a short polyproline-rich region previously named CHPPR (chondrocyte protein with a polyproline region). Mtfr1 targets mitochondria and is mostly associated with the mitochondrial inner membranes.^12^ Recent finding suggests that Mtfr1-expressing cells reveal the presence of a number of small spheroid mitochondria which indicates that Mtfr1 is able to promote mitochondrial fission.^12^ In addition, study also reports that deficiency of Mtfr1 results in oxidative DNA damage.^13^ However, it is not yet clear whether Mtfr1 participates in the regulation of mitochondrial dynamics in cardiomyocytes.

MicroRNAs (miRNAs) are a class of short single-stranded noncoding endogenous RNAs that suppress protein expression by binding to mRNAs.^14^ MiRNAs were identified as important transcriptional and post-transcriptional inhibitors of gene expression. Numerous studies have suggested that miRNAs are involved in several fundamental cellular processes, such as cell survival, apoptosis, necrosis and development.^3, 4, 15, 16^ Gain or loss function studies have uncovered important roles for miRNAs in cardiac diseases, including myocardial infarction, cardiac hypertrophy and heart failure.^17^ During cardiac diseases, miRNA-expression profile is significantly changed, indicating an extremely dynamic regulation of miRNAs in the adult heart.^18^ Although the function of miRNAs has been widely studied, few works have been focused on miRNAs in the mitochondrial network regulation.

NFATc3, also named NFAT4, is produced by *NFAT4* gene located on murine chromosome 8.^19^ NFAT4 is modulated by Ca^2+^/calmodulin signaling pathway and its stimulation requires calcium oscillation.^20^ NFAT4 contains a conserved rel similarity domain and an SP repeat region.^19^ Overexpression of NFAT4 activates NFAT site-dependent transcription and regulates distinct subset of genes.^19^ It has been proved that NFAT4 are also expressed in the heart and is a downstream target of calcineurin.^21^ In the quiescent cells, NFAT4 is retained in the cytoplasm.^22^ After dephosphorylation by calcineurin, NFAT4 is activated and imported into the nucleus.^22^ The previous work shows that NFAT4 has an essential role in reducing voltage-gated K^+^ currents after a myocardial infarction.^23^ However, the role of NFAT4 in regulating cardiomyocyte mitochondrial fission and apoptosis remains largely unveiled.

Our present study unveils that Mtfr1 is involved in the regulation of mitochondrial network in cardiomyocytes. Knockdown of Mtfr1 inhibits mitochondrial fission and apoptosis in cardiomyocytes. Also, knockdown of Mtfr1 in mice exhibit a reduced myocardial infarction sizes upon myocardial ischemia/reperfusion (I/R) injury *in vivo*. In search for the mechanisms by which Mtfr1 is upregulated under a pathological condition, we identified miR-324-5p as a translational inhibitor of Mtfr1. MiR-324-5p inhibits mitochondrial fission, apoptosis and myocardial infarction through downregulating Mtfr1. We further find that NFAT4 transcriptionally suppresses miR-324-5p expression and thus regulates mitochondrial fission and apoptosis. Our results reveal a novel pathway in which NFAT4 promotes Mtfr1 expression by suppressing miR-324-5p transcriptional activity, which could contribute to the regulation of mitochondrial dynamics and apoptosis.

## Results

### Mtfr1 regulates mitochondrial fission and apoptosis in cardiomyocytes

**Figure 1:**
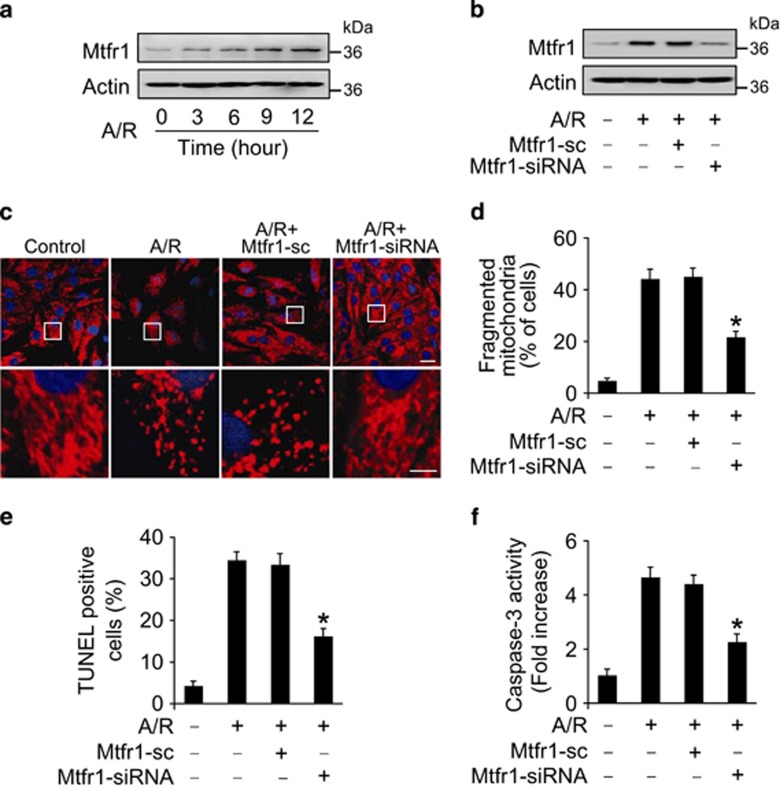
Mtfr1 regulates mitochondrial fission and apoptosis *in vitro*. (**a**) A/R induces an increase in Mtfr1 levels. Cardiomyocytes were treated with A/R at indicated time. Mtfr1 levels were analyzed by immunoblot. (**b**) Knockdown of Mtfr1 attenuates the increased Mtfr1 levels upon A/R treatment. Cardiomyocytes were infected with adenoviral constructs of Mtfr1 siRNA or its scrambled form (sc) and then exposed to A/R. Mtfr1 levels were analyzed by immunoblot. (**c** and **d**) Knockdown of Mtfr1 attenuates mitochondrial fission induced by A/R treatment. Cardiomyocytes were infected with adenoviruses harboring Mtfr1 siRNA or its sc form and then treated with A/R. Mitochondria were stained by MitoTracker red and the nuclei were visualized by DAPI. (**c**) Scale bar, 20 *μ*m (upper panel); scale bar, 5 *μ*m (lower panel). The cells with fragmented mitochondria were counted. (**d**) *P<0.05 *versus* A/R alone. (**e** and **f**) Knockdown of Mtfr1 reduces A/R-induced apoptosis. Cardiomyocytes were infected with adenoviruses harboring Mtfr1 siRNA or its sc form and then were subjected to 2-h anoxia followed by 12-h reoxygenation. TUNEL was employed to analyze apoptotic cells. TUNEL-positive cells were counted and calculated. (**e**) The caspase-3 activity was analyzed by using an Apo-ONE Homogeneous Caspase-3/7 assay kit (Madison, WI, USA) (**f**) *P<0.05 *versus* A/R alone

To test whether Mtfr1 participates in the regulation of mitochondrial fission in cardiomyocytes, we treated cardiomyocytes with A/R to induce mitochondrial fission and apoptosis. A/R treatment induced the increase of Mtfr1 expression levels (Figure 1a). We then tested whether Mtfr1 is involved in the occurrence of mitochondrial fission. Knockdown of Mtfr1 by siRNA technology efficiently attenuated A/R-induced Mtfr1 levels (Figure 1b). Reduced mitochondrial fission as revealed by the analysis of mitochondrial morphology (Figure 1c) and the counting of the cells with fission (Figure 1d and Supplementary Figure 1A) were also observed together with Mtfr1 downregulation. Further, knockdown of Mtfr1 attenuated A/R-induced apoptosis analyzed by TUNEL assay (Figure 1e and Supplementary Figure 1B) and Caspase 3 activity (Figure 1f). Taken together, these results suggest that Mtfr1 participates in the regulation of mitochondrial fission and apoptosis in cardiomyoctyes.

### Mtfr1 regulates mitochondrial fission and apoptosis *in vivo*

**Figure 2:**
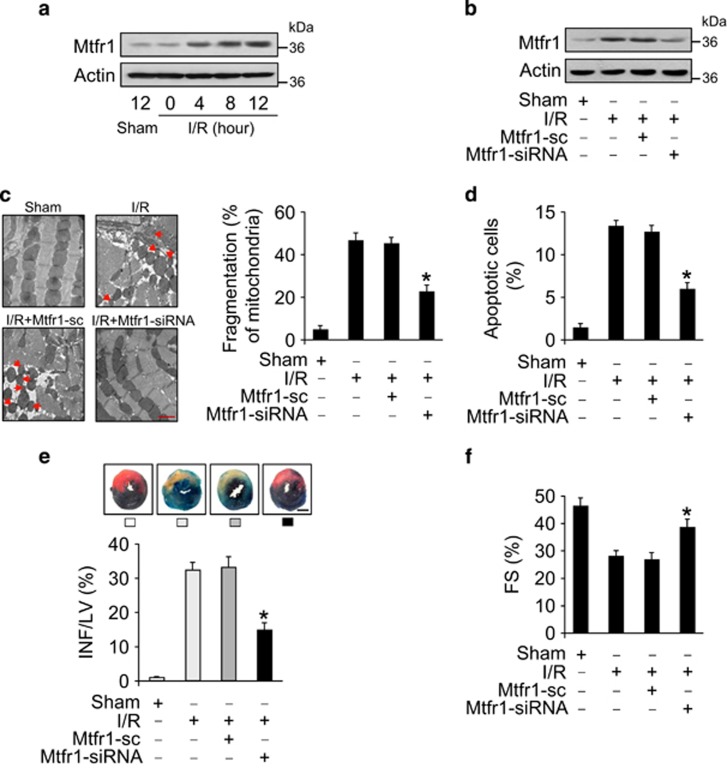
Mtfr1 regulates mitochondrial fission and apoptosis *in vivo*. (**a**) Mtfr1 is upregulated in response to ischemia/reperfusion. Mice were subjected to various time periods of ischemia, and Mtfr1 levels were detected by immunoblot. (**b**) Mtfr1 siRNA can efficiently knockdown Mtfr1 in the animal model. After intracoronary delivery of adenoviruses harboring Mtfr1 siRNA or the scrambled form, the mice were subjected to I/R injury. Mtfr1 levels were detected by immunoblot. (**c** and **d**) Knockdown of Mtfr1 attenuates I/R-induced mitochondrial fission and apoptosis. After intracoronary delivery of adenoviruses harboring Mtfr1 siRNA or the scrambled form, the mice were subjected to 45-min ischemia, then 3-h reperfusion. Mitochondrial fission was analyzed. (**c**) TUNEL assay was employed to detect apoptotic cells. (**d**) *P<0.05 *versus* I/R alone. (**e**) Knockdown of Mtfr1 decreases myocardial infarct sizes in response to I/R. Mice were treated as described for (**b**) and infarct sizes were calculated. The upper panels are myocardial representative photos of mid-ventricular myocardial slices. Scale bar, 2 mm. *P<0.05 *versus* I/R alone. (**f**) Echocardiographic analysis. Mice were treated as described for (**b**) and echocardiography was employed to test heart function. Fractional shortening (FS) were calculated. *P<0.05 *versus* I/R alone

To understand the pathophysiological role of Mtfr1, we detected whether Mtfr1 was involved in the pathogenesis of myocardial infarction in the animal model. We employed the myocardial I/R model and found that I/R induced an elevation in Mtfr1 expression levels (Figure 2a). The administration of Mtfr1 siRNA adenoviruses *in vivo* reduced the elevated expression of Mtfr1 upon I/R injury (Figure 2b). Knockdown of Mtfr1 also resulted in a reduction in mitochondrial fission (Figure 2c), apoptosis (Figure 2d) and infarct size (Figure 2e) upon I/R. Echocardiography showed that the cardiac function was ameliorated in Mtfr1-knockdown group in response to I/R injury (Supplementary Figures 1C and D, and Figure 2f). These data suggest that Mtfr1 participates in mediating the signal for mitochondrial fission and apoptosis in the heart.

### miR-324-5p participates in the regulation of Mtfr1 expression

**Figure 3:**
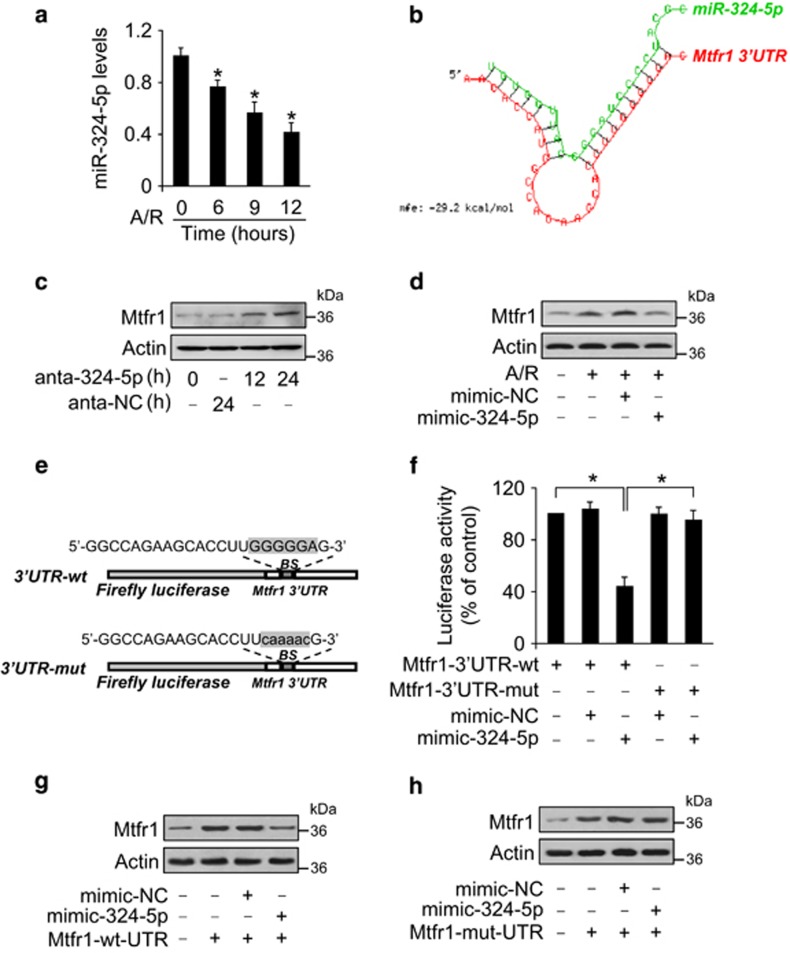
MiR-324-5p participates in the regulation of Mtfr1 expression. (**a**) MiR-324-5p levels were analyzed by qRT-PCR. *P<0.05 *versus* control. (**b**) Analysis of Mtfr1 3′UTR potential binding site for miR-324-5p. (**c**) Knockdown of miR-324-5p induces an elevated Mtfr1 levels. Cardiomyocytes were transfected with antagomir miR-324-5p (anta-324-5p) or antagomir-negative control (anta-NC). The expression of Mtfr1 was detected by immunoblot. (**d**) miR-324-5p attenuates A/R-induced Mtfr1 upregulation. Cardiomyocytes were transfected with miR-324-5p mimic (mimic-324-5p) and its negative control (mimic-NC), and then were treated with A/R. Mtfr1 levels were detected by immunoblot. (**e**) miR-324-5p binding site was mutated in the Mtfr1 3′UTR. (**f**) miR-324-5p inhibits the luciferase activity of Mtfr1 with wild-type 3′UTR. HEK293 cells were transfected with Mtfr1 with a wild-type 3′UTR or mutated 3′UTR, miR-324-5p mimic or its negative control, and then the cells were harvested, and luciferase activity was measured; *P<0.05. (**g** and **h**) miR-324-5p inhibits the expression of Mtfr1 with wild-type 3′UTR. Cardiomyocytes were infected with adenoviral Mtfr1 with a wild-type 3′UTR or mutated 3′UTR, and then were transfected with miR-324-5p mimic. The expression of Mtfr1 was assayed by immunoblot

miRNAs are a class of small noncoding RNAs and act as negative regulators of gene expression. To explore the underlying mechanism by which Mtfr1 is upregulated upon A/R and I/R, we tested whether Mtfr1 can be regulated by miRNA. We first screened some cardiac-associated miRNAs that had been reported in past several years. Among several miRNAs, miR-324-5p levels were significantly downregulated upon A/R (Figure 3a) and other miRNAs remain unchanged (data not shown). We also analyzed the 3′UTR of Mtfr1 using the RNAhybrid program and observed that Mtfr1 is a potential target of miR-324-5p (Figure 3b), which promoted us to focus on the function of miR-324-5p. We then explored whether Mtfr1 expression can be regulated by miR-324-5p. We found that miR-324-5p had no effect on the mRNA level of Mtfr1 (Supplementary Figures 2A and B), whereas knockdown of endogenous miR-324-5p by antagomir induced an increase in Mtfr1 protein levels (Figure 3c), and enforced expression of miR-324-5p attenuated the increase of Mtfr1 protein levels in response to A/R treatment (Figure 3d). These results indicate that miR-324-5p can specifically regulate Mtfr1.

Next, we employed the luciferase assay system to test whether miR-324-5p can influence the translation of Mtfr1. As shown in Figure 3f, the luciferase reporter assay revealed that the wild-type (wt) 3′-UTR of Mtfr1 exhibited a low translation level in the presence of miR-324-5p, whereas the mutated 3′-UTR (Figure 3e) did not show a significant response to miR-324-5p. We finally tested whether miR-324-5p miR-324-5p regulates Mtfr1 expression by targeting its 3′UTR. Mtfr1 with wt 3′UTR was expressed at a low level in the presence of miR-324-5p (Figure 3g). Introduction of mutations into the miR-324-5p binding site abolished the inhibitory effect of miR-324-5p on Mtfr1 expression (Figure 3h). Taken together, these results suggest that Mtfr1 is a specific target of miR-324-5p.

### miR-324-5p inhibits mitochondrial fission and apoptotic program

**Figure 4:**
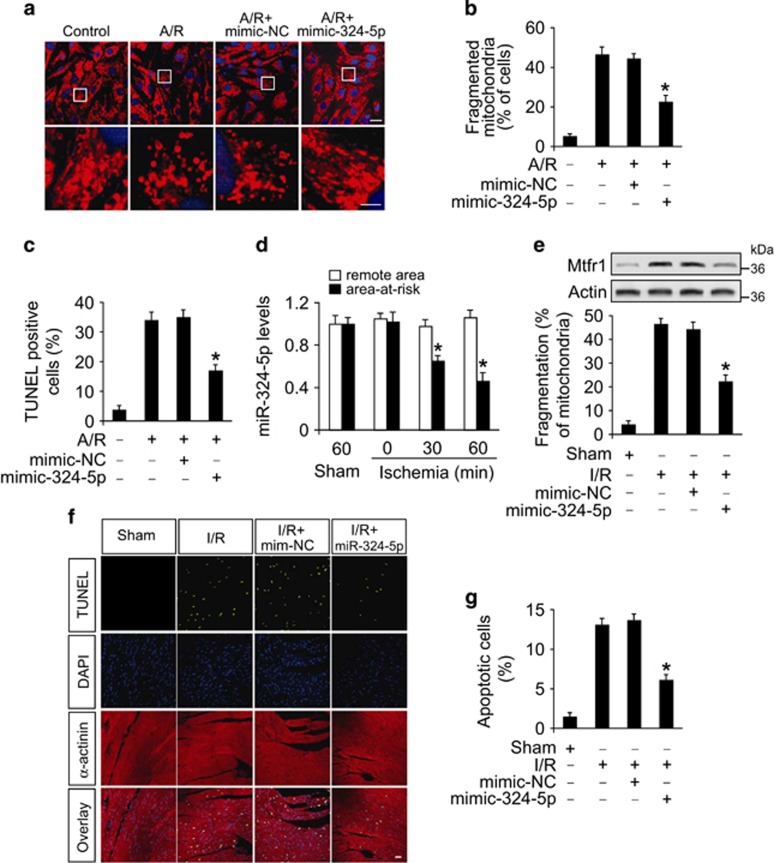
MiR-324-5p inhibits mitochondrial fission and apoptosis in cardiomyocytes and *in vivo*. (**a** and **b**) miR-324-5p suppresses A/R-induced mitochondrial fission. Cardiomyocytes were transfected with miR-324-5p mimic or NC. After that, they were treated with A/R. Mitochondrial fission was assayed. (**a**) Scale bar, 20 *μ*m (upper panel); scale bar, 5 *μ*m (lower panel). *P<0.05 *versus* A/R alone. (**c**) MiR-324-5p suppresses A/R-induced apoptosis. Cardiomyocytes were transfected with miR-324-5p mimic or NC and were treated with A/R. Apoptosis was assayed by TUNEL. *P<0.05 *versus* A/R alone. (**d**) Analysis of miR-324-5p expression levels. Mice were subjected to I/R at indicated time and the expression of miR-324-5p was assayed by qRT-PCR. *P<0.05 *versus* sham or 0-min group. (**e**) MiR-324-5p attenuates Mtfr1 expression and mitochondrial fission upon I/R injury. Adult C57BL/6 mice received miR-324-5p mimic or mimic-NC for 3 days. They were then subjected to I/R. Mtfr1 levels were analyzed by immunoblot (upper panel). The percentage of mitochondrial fission was calculated (lower panel). *P<0.05 *versus* I/R alone. (**f** and **g**) MiR-324-5p attenuates apoptosis upon I/R injury. Mice were treated as described in **e**. Apoptosis was detected by TUNEL assay. TUNEL-positive cardiomyocyte nuclei (apoptotic cells) are green. Nuclei stained by DAPI showed blue. Cardiomyocytes were labeled with *α*-actinin. *P<0.05 *versus* I/R alone

We explored the functional role of miR-324-5p in mitochondrial fission and apoptosis. Enforced expression of miR-324-5p efficiently increased the miR-324-5p levels in response to A/R treatment (Supplementary Figure 2C), and miR-324-5p also attenuated A/R-induced mitochondrial fission (Figures 4a and b). Concomitantly, apoptosis was reduced in the presence of miR-324-5p as revealed by TUNEL assay (Figure 4c) and Caspase 3 activity (Supplementary Figure 2D). These data indicate that miR-324-5p can inhibit mitochondrial fission and apoptosis in cardiomyocytes.

Subsequently, we detected whether miR-324-5p is involved in the pathogenesis of myocardial infarction in the animal model. MiR-324-5p levels were reduced in response to an I/R injury (Figure 4d). Enforced expression of miR-324-5p by mimic delivery *in vivo* resulted in a reduction in Mtfr1 expression (Figure 4e, upper panel), mitochondrial fission (Figure 4e, lower panel) and apoptosis (Figures 4f and g). We then tested whether miR-324-5p affects the size of myocardial infarction and cardiac function. Administration of miR-324-5p mimics significantly reduced infarct sizes upon I/R injury (Supplementary Figure 3A). Concomitantly, the heart function was ameliorated when the mice were treated with miR-324-5p mimics as assessed by echocardiography (Supplementary Figures 3B and D).

### miR-324-5p regulates mitochondrial fission and apoptosis through targeting Mtfr1

**Figure 5:**
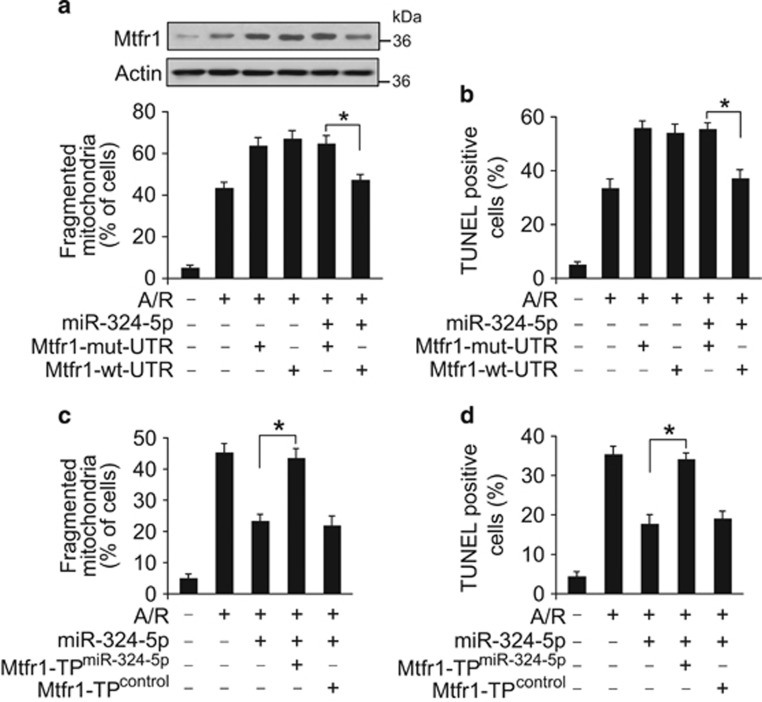
MiR-324-5p regulates mitochondrial fission and apoptosis through Mtfr1. (**a** and **b**) MiR-324-5p suppresses the expression of Mtfr1, mitochondrial fission and apoptosis in the presence of Mtfr1 with wild-type 3′UTR rather than mutated 3′UTR. Cardiomyocytes were infected with adenoviral Mtfr1-wt-UTR or Mtfr1-mut-UTR and were transfected with miR-324-5p mimic. After transfection, cells were treated with A/R. Mtfr1 levels were detected by immunoblot (**a**, upper panel). Mitochondrial fission (**a**, lower panel) and apoptosis (**b**) were assayed. *P<0.05. (**c** and **d**) The inhibitory effect of miR-324-5p on mitochondrial fission and apoptosis is abolished by Mtfr1 target protector. Cardiomyocytes were co-transfected with miR-324-5p mimic, Mtfr1 target protector (Mtfr1-TP^miR-324-5p^) or the protector control (Mtfr1-TP^control^), and then were treated with A/R. Mitochondrial fission (**c**) and apoptosis (**d**) were detected. *P<0.05

We explored how miR-324-5p exerts its effect on the mitochondrial network. As miR-324-5p is able to suppress Mtfr1 expression, we thus tested whether Mtfr1 is a mediator of miR-324-5p. Mtfr1 with mutated 3′UTR, but not the wt Mtfr1, attenuates the inhibitory effect of miR-324-5p on Mtfr1 expression (Figure 5a, upper panel), mitochondrial fission (Figure 5a, lower panel) and apoptosis (Figure 5b). To further confirm the relationship between miR-324-5p and Mtfr1 in mitochondrial fission machinery, we employed the target protector technology in which a target protector is able to disrupt the specific interaction of miRNA–mRNA pairs. To this end, we produced Mtfr1 target protector and observed that the inhibitory effect of miR-324-5p on mitochondrial fission and apoptosis was reduced in the presence of the Mtfr1 target protector (Figures 5c and d). These data suggest that miR-324-5p targets Mtfr1 in the cascades of mitochondrial fission and apoptosis.

### NFAT4 regulates mitochondrial fragmentation and apoptosis by affecting miR-324-5p transcription

**Figure 6:**
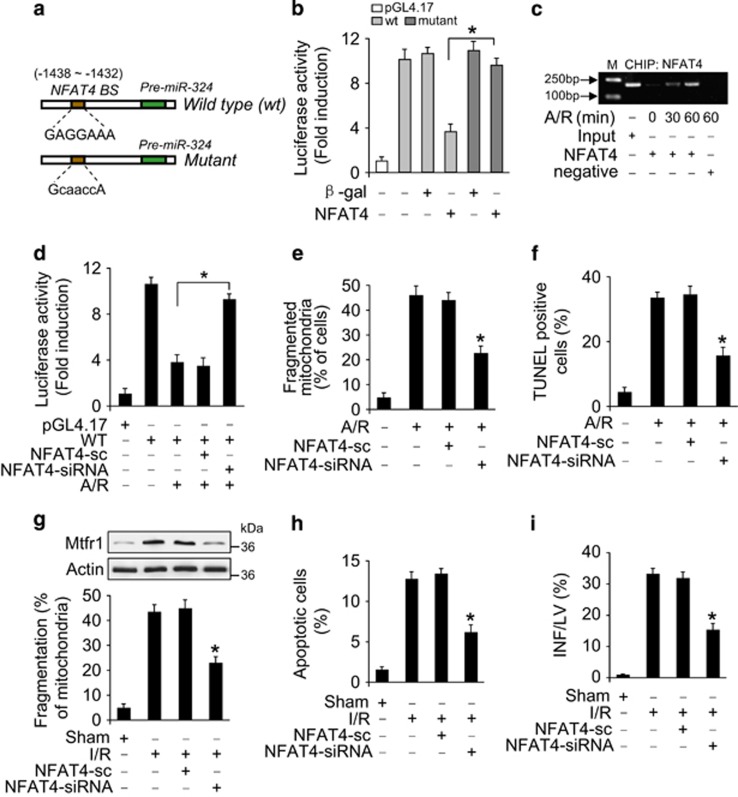
miR-324-5p is a transcriptional target of NFAT4. (**a**) Mouse miR-324-5p promoter region contains a potential NFAT4 binding site. (**b**) NFAT4 attenuates miR-324-5p promoter activity. Cardiomyocytes were treated with the adenoviral *β*-gal or NFAT4, the constructs of the empty vector (pGL-4.17), the wild-type promoter (wt) or the promoter with mutations in the binding site (mutant), respectively. Luciferase activity was assayed. *P<0.05. (**c**) ChIP analysis of NFAT4 binding to the promoter of miR-324-5p. (**d**) Knockdown of NFAT4 inhibits the decrease of miR-324-5p promoter activity induced by A/R. Cardiomyocytes were treated with the adenoviral NFAT4-siRNA or NFAT4-sc, the constructs of the empty vector (pGL-4.17), the wt promoter, then were treated with A/R. Luciferase activity was assayed. *P<0.05. (**e** and **f**) Knockdown of NFAT4 reduces mitochondrial fission and apoptosis. Cardiomyocytes were infected with adenoviral NFAT4-siRNA or NFAT4-sc, and then exposed to A/R, mitochondrial fission (**e**) and apoptosis (**f**) were analyzed. *P<0.05 *versus* A/R alone. (**g** and **h**) Knockdown of NFAT4 attenuates mitochondrial fission and apoptosis upon I/R. After intracoronary delivery of adenoviruses harboring NFAT4 siRNA or the scrambled form, the mice were subjected to I/R injury. Mtfr1 levels were analyzed by immunblot (**g**, upper panel). Mitochondrial fission (**g**, lower panel) and apoptosis (**h**) were analyzed. *P<0.05 *versus* I/R alone. (**i**) Knockdown of NFAT4 decreases myocardial infarct sizes in response to I/R. Mice were treated as described for (**g**), and infarct sizes were calculated. *P<0.05 *versus* I/R alone

We explored how miR-324-5p expression is regulated under a pathological condition. MiRNA could be regulated at the transcriptional level as we described previously.^3^ Thus, we analyzed the promoter region of mouse miR-324-5p and observed that there was a potential binding site of NFAT4 (Figure 6a). The luciferase assay demonstrated that NFAT4 attenuated the wt miR-324-5p promoter activity (Figure 6b). However, mutations in the NFAT4 binding site abolished the effect of NFAT4 on miR-324-5p promoter activity (Figure 6b). Enforced expression of NFAT4 (Supplementary Figure 4A) induced a decrease in miR-324-5p levels (Supplementary Figure 4B), whereas NFAT4 knockdown (Supplementary Figure 4C) exhibited an increased expression levels of miR-324-5p (Supplementary Figure 4D). The ChIP assay revealed that NFAT4 is bound to the miR-324-5p promoter under the physiological condition. A/R treatment led to an increase in the association levels of NFAT4 with miR-324-5p promoter (Figure 6c), suggesting that miR-324-5p is a potential transcriptional target of NFAT4. Furthermore, A/R induced a decrease in miR-324-5p promoter activity in cardiomyocytes (Figure 6d), and knockdown of NFAT4 attenuated the inhibitory effect of A/R on miR-324-5p promoter activity (Figure 6d). These data indicate that miR-324-5p can be transcriptionally inhibited by NFAT4.

We investigated the role of NFAT4 in mitochondrial fission and apoptosis in cardiomyocytes and *in vivo*. Enforced expression of NFAT4 induced an increase in Mtfr1 levels (Supplementary Figure 5A), whereas NFAT4 knockdown exhibited a decreased Mtfr1 expression (Supplementary Figure 5B). A/R induced an elevation in NFAT4 expression level (Supplementary Figure 6A). Knockdown of NFAT4 attenuated NFAT4 expression (Supplementary Figure 6B), mitochondrial fission (Figure 6e and Supplementary Figure 6C) and apoptosis (Figure 6f and Supplementary Figure 6D) upon A/R. Furthermore, NFAT4 knockdown *in vivo* (Supplementary Figure 7A) reduced Mtfr1 expression (Figure 6g, upper panel) and induced the increase of miR-324-5p levels (Supplementary Figure 7B). Knockdown of NFAT4 attenuated mitochondrial fission (Figure 6g, lower panel), apoptosis (Figure 6h) and myocardial infarction size (Figure 6i) upon I/R injury. These data suggest that NFAT4 participates in mediating the signal for mitochondrial fission and apoptosis in the heart.

## Discussion

Recently, it has been found that mitochondria has an important role in regulating cell death. Mitochondria constantly fuse and divide to form a dynamic network.^24^ Mitochondria fragmentation during cell death has been shown to have a key role in cell death progression, including release of the mitochondrial apoptotic proteins. Other morphological changes, such as cristae remodeling, swelling and outer membrane rupturing, are also involved in cell death initiation.^24^ Here we present evidence for a critical role of Mtfr1 in regulating mitochondrial dynamics. Mtfr1 can regulate mitochondrial fission and apoptosis in cardiomyocytes and knockdown of Mtfr1 in mice showed a reduced myocardial infarction sizes upon ischemia injury *in vivo*. In addition, we demonstrated that miR-324-5p is responsible for the downregulation of Mtfr1, and modulation of miR-324-5p levels also affects mitochondrial fission, apoptosis and myocardial infarction. MiR-324-5p exerts its effect on mitochondrial fission and apoptosis through targeting Mtfr1. We further identify that NFAT4 regulates mitochondrial fission and apoptosis by suppressing miR-324-5p in transcriptional level. Our results provide a novel evidence demonstrating that NFAT4, miR-324-5p and Mtfr1 constitute an axis in the regulated machinery of mitochondrial network and apoptosis.

The present work shows that Mtfr1 participates in the regulation of mitochondrial fission in cardiomyocytes. This is in consistence with other reports demonstrating the promotive effect of Mtfr1 on mitochondrial fission. The function of Mtfr1 in apoptosis remains unknown. Our work for the first time suggests that Mtfr1 participates in the regulation of apoptosis in cardiomyocytes. Knockdown of Mtfr1 in mice exhibits a reduced mitochondrial fission, apoptosis and myocardial infarction sizes upon I/R injury. Whether Mtfr1 can also act as a mediator of mitochondrial fission and apoptosis in other cell types need to be further clarified in our future research.

Some miRNAs have been reported to regulate apoptosis in cardiomyocytes.^25, 26, 27^ However, few works have been focused on miRNAs in the mitochondrial network regulation. Our present work for the first time demonstrates that miR-324-5p regulates mitochondrial fission and apoptosis both *in vitro* and *in vivo* by targeting Mtfr1. MiR-324-5p and Mtfr1 have complementary expression patterns during cardiomyocytes apoptosis and myocardial infarction. Overexpression of miR-324-5p resulted in decreased Mtfr1 expression levels, mitochondrial fission, cardiomyocytes apoptosis and myocardial infarction. Accordingly, miR-324-5p might be a potential therapeutic target for myocardial infarction. Our results further support the notion that miRNAs are important modifiers of gene expression program in cardiovascular diseases.

Many scientific works report that NFAT4 has an important role in cardiomyocytes. For example, the nuclear occupancy of NFAT4 was increased in heart failure.^28^ Inhibiting the calcineurin-NFAT pathway protects the heart from excessive cardiac remodeling.^29^ NFAT4 activation also contributes to hypertension and increased wall thickness in the heart.^30^ Our present work for the first time reveals the function of NFAT4 in cardiomyocyte mitochondrial fission and apoptosis. Our results show that knockdown of NFAT4 inhibits mitochondrial fission and apoptosis in cardiomyocytes. Knockdown of NFAT4 in mice also demonstrate reduced mitochondrial fission and myocardial infarction size upon I/R injury. NFAT4 affects the mitochondrial dynamics and apoptotic pathway by regulating the expression of miR-324-5p. It would be interesting to study the role of other targets of NFAT4 in the pathway of mitochondrial fission and apoptosis. Modulation of their levels may provide a new approach for tackling myocardial infarction.

Hitherto, extraordinary efforts have been devoted to determining the molecular and pathophysiological characteristics of the diseased heart, aiming to develop novel diagnostic and therapeutic strategies to combat cardiac diseases. Our study suggests a potential signal pathway of miR-324-5p and Mtfr1 for cardiac diseases and they will be intriguing targets for therapeutic intervention.

## Materials and methods

### Adenoviral constructions and infection

The mouse Mtfr1 was synthesized by PCR using mouse cDNA as the template. The adenovirus harboring the Mtfr1 was constructed using the Adeno-X expression system (Clontech, Otsu, Japan). The adenovirus containing *β*-galactosidase (*β*-gal) is as we described elsewhere.^31^ The mouse Mtfr1 RNA interference (siRNA) target sequence is 5′-CCATATGGTTCATCTCGAA-3′. A scramble form was used as a control, 5′-TACTATCGTCACTGACTAG-3′. The mouse Mtfr1-siRNA-B target sequence is 5′-TGAGCTTGCTGCCCTTAGA-3′. A scramble form was used as a control, 5′-GCTGAGTTGACCTCTATCG-3′. The NFAT4 RNA interference target sequence is 5′-AAATGTCAAGGGGCTCACA-3′. A scramble form was used as a control, 5′-ACTCACGTGACGAGAGATA-3′. The mouse NFAT4-siRNA-B target sequence is 5′-CCCTTTGAGTGCCCAAGTA-3′. A scramble form was used as a control, 5′-ATTCGCCTAGCTGACTGAC-3′. The adenoviruses harboring siRNA or their scramble form were constructed using the pSilencer adeno 1.0-CMV System (Ambion, Grand Island, NY, USA) according to the Kit's instructions. All constructs were amplified in HEK293 cells. Adenoviral infection of cardiomyocytes was performed as we described previously.^32^

### Transfection of the antagomir

The chemically modified antagomir complementary to miR-324-5p designed to inhibit endogenous miR-324-5p expression, the antagomir-negative control (antagomir-NC) were obtained from GenePharma Co. Ltd. (Shanghai, China). The miR-324-5p antagomir sequence was 5′-ACACCAAUGCCCUAGGGGAUGCG-3′. The antagomir-NC sequence was 5′-CAGUACUUUUGUGUAGUACAA-3′. Cells were transfected with the antagomirs or the antagomir-NC using Lipofectamine 2000 (Invitrogen, Grand Island, NY, USA) according to the manufacturer's instruction.

### Target protector preparation and transfection

Target protector was designed and named as others and we described,^31^ In brief, Mtfr1-TP^miR-324-5p^ sequence is 5′- CTCCCCCAAGGTGCTTCTGGCCATG-3′. Mtfr1-TP^control^ sequence is 5′-TGACAAATGAGACTCTCTCCTCTCC-3′. They were synthesized by Gene Tools, and were transfected into the cells using the Endo-Porter kit (Gene Tools, Philomath, OR, USA) according to the kit's instructions.

### Constructions of mouse miR-324-5p promoter

The promoter of miR-324-5p was amplified from mouse genome using PCR. The forward primer was 5′-GCTATCACAGAGCATTTTCTCAT-3′. The reverse primer was 5′-TGCACCAAACACGACTTTTAACC-3′. The promoter fragment was finally cloned into the vector pGL4.17 (Promega, Madison, WI, USA). The introduction of mutations in the putative NFAT4 binding site was performed with the QuikChange II XL Site-Directed Mutagenesis Kit (Stratagene, La Jolla, CA, USA) using the wt vector as a template.

### Animal experiments

Male adult C57BL/6 mice (8 weeks old) were obtained from Institute of Laboratory Animal Science of Chinese Academy of Medical Sciences (Beijing, China). All experiments were performed according to the protocols approved by the Institute Animal Care Committee. The mice received on three consecutive days, intravenous injections of miR-324-5p mimic, or its control at a dose of 35 mg/kg body weight in a small volume (0.2 ml) per injection. To perform intracoronary delivery of adenovirus, 5 days before the I/R operation, the mice were anesthetized. The chest was opened and Mtfr1-siRNA adenoviruses (2 × 10^11^ MOI) or NFAT4-siRNA adenoviruses (2 × 10^10^ MOI) were injected with a catheter from the apex of the left ventricle into the aortic root while the aorta and pulmonary arteries were cross-clamped. The clamp was maintained for 20 s when the heart pumped against a closed system. The chest was closed and the animal was transferred back to its cage for recovery.

To perform I/R surgery, the mice were treated as we previously described.^25^ All mice were subjected to 45-min ischemia and then 3-h reperfusion. Sham-operated group experienced the same procedure except the snare was left untied. Cardiac function of these groups of animals was evaluated by echocardiographic analysis 14 days after the surgery. Evans blue dye was treated as described,^25^ The areas of infarction (INF) and nonischemic left ventricle (LV) were assessed with computer-assisted planimetry (NIH Image 1.57) by an observer blinded to the sample identity. The ratio of INF/LV was calculated as described.^25^

### Statistical analysis

Data are expressed as the mean±S.E.M. of at least three independent experiments. We used a one-way analysis of variance for multiple comparisons. A value of *P*<0.05 was considered significant.

## Abbreviations

miRNAs: microRNAs
A/R: Anoxia/reoxygenation
I/R: Ischemia/reperfusion
Mtfr1: Mitochondrial fission regulator 1
TUNEL: Terminal deoxynucleotidyl transferase-mediated dUTP nick-end-labeling
EM: Electron microscopy
qRT-PCR: Quantitative reverse transcription polymerase chain reaction
TP: Target protector
