# Supplementary material for: NFAT4-dependent miR-324-5p regulates mitochondrial morphology and cardiomyocyte cell death by targeting Mtfr1
Source: Cell Death Dis. 2015 Dec 3;6(12):e2007–. doi: 10.1038/cddis.2015.348 (PMC4720883; doi:10.1038/cddis.2015.348)
Supplement: Supplementary Information [file cddis2015348x1.doc]

**Supplementary Methods**

**Cardiomyocyte culture and treatment**-Cardiomyocytes were isolated from 1-2 days old mice as we described [^1^](#_ENREF_1), Briefly, after dissection hearts were washed, minced in HEPES-buffered saline solution. Tissues were then dispersed in a series of incubations at 37°C in HEPES-buffered saline solution containing 1.2 mg/ml pancreatin and 0.14 mg/ml collagenase (Worthington). After centrifugation cells were re-suspended in Dulbecco’s modified Eagle medium/F-12 (GIBCO) containing 5% heat-inactivated horse serum, 0.1 mM ascorbate, insulin-transferring-sodium selenite media supplement (Sigma, St. Louis, MO), 100 U/ml penicillin, 100 μg/ml streptomycin, and 0.1 mM bromodeoxyuridine. The dissociated cells were pre-plated at 37°C for 1 h. The cells were then diluted to 1x10^6^ cells/ml and plated in 10 μg/ml laminin-coated different culture dishes according to the specific experimental requirements. Anoxia/reoxygenation was performed as we described elsewhere [^2^](#_ENREF_2). Briefly, cells were placed in an anoxic hamber with a water-saturated atmosphere composed of 5% CO2 and 95% N2. After 2h anoxia, the cells were subjected to reoxygenation (95% O2 and 5% CO2) for 12h.

**Apoptosis assays**-Apoptosis was determined by the terminal deoxyribonucleotidyl transferase–mediated TUNEL using a kit from Roche. The detection procedures were in accordance with the kit instructions. Caspase-3 was determined by analyzing its activity using an Apo-ONE® Homogeneous Caspase-3/7 assay kit from Promega according to the manufacturer’s protocol.

**Immunoblot -**Immunoblot was carried out as we previously described [^3^](#_ENREF_3). Briefly, the cells were lysed for 1 h at 4 °C in a lysis buffer (20 mmol/L Tris pH 7.5, 2 mmol/L EDTA, 3 mmol/L EGTA, 2 mmol/L dithiothreitol (DTT), 250 mmol/L sucrose, 0.1 mmol/L phenylmethylsulfonyl fluoride, 1% Triton X-100) containing a protease inhibitor cocktail. The samples were subjected to 12% SDS-PAGE and transferred to nitrocellulose membranes. Equal protein loading was controlled by Ponceau Red staining of membranes. Blots were probed using the primary antibodies. The anti-Mtfr1 and anti-Actin were from Abcam. The anti-NFAT4 was from Santa Cruz Biotechnology. After four times washing with PBS, the horseradish peroxidase-conjugated secondary antibodies were added. Antigen-antibody complexes were visualized by enhanced chemiluminescence.

**Quantitative reverse transcription-PCR (qRT-PCR)-**Stem-loop qRT-PCR for mature miR-324-5p was performed as described on a CFX96 Real-Time PCR Detection System (Bio-Rad). Total RNA was extracted using Trizol reagent. After DNAse I (Takara, Japan) treatment, RNA was reverse transcribed with reverse transcriptase (ReverTra Ace, Toyobo). The levels of miR-324-5p analyzed by qRT-PCR were normalized to that of U6. U6 primers were forward: 5’-GCTTCGGCAGCACATATACT-3’; reverse: 5’-AACGCTTCACGAATTTGCGT-3’.

**Chromatin immunoprecipitation (ChIP) assay***-*ChIP assay was performed as we described [^4^](#_ENREF_4). The purified DNA was used as a template and amplified with the following primer sets: For the analysis of NFAT4 binding to the promoter region of miR-324-5p, the oligonucleotides were as follows: forward: 5’-GCTATCACAGAGCATTTTCTCAT-3’; reverse: 5’-TATGCCATGGTGGTGGTTCTGT-3’.

**Preparations of the luciferase construct of Mtfr1 3’UTR and luciferase activity assay**-Mtfr1 3’UTR was amplified by PCR. The forward primer was 5’-CTGTAGTGTTCATTGGTCTCCTT-3’; the reverse primer was 5’- GCTTGACACAGCTAGAGTCATC-3’. To produce mutated 3’UTR, the mutations were generated using QuikChange II XL Site-Directed Mutagenesis Kit (Stratagene). The constructs were sequence verified. Wild type and mutated 3’UTRs were subcloned into the pGL3 vector (Promega) immediately downstream of the stop codon of the luciferase gene.

Luciferase activity assay was performed using the Dual-Luciferase Reporter Assay System (Promega) according to the manufacturer’s instructions. Cells were co-transfected with the plasmid constructs of 150 ng/well of pGL3-Mtfr1-3’UTR or pGL3-Mtfr1-3’UTR-mut using Lipofectamine 2000 (Invitrogen), then were infected with adenovirus miR-324-5p or β-gal at a moi of 80. At 48 h after infection, luciferase activity was measured.

**Mitochondrial staining**-Mitochondrial staining was carried as we and others described with modifications . Briefly, cells were plated onto the cover-slips coated with 0.01% poly-L-lysine. After treatment they were stained for 20 min with 0.02 μM MitoTracker Red CMXRos (Molecular Probes). Mitochondria were imaged using a laser scanning confocal microscope (Zeiss LSM510 META). The percentage of cells with fragmented mitochondria relative to the total number of cells is presented as the mean ± SEM of at least three independent experiments, counted by an observer blinded to the experimental conditions. Six distinct fields for each 50 cells were counted. At least 300 cells per group were counted.

**Electron Microscopy-**Heart ultrastructural analyses were performed to quantify mitochondrial fission. Sample preparations and conventional EM were carried out as described before [^2^](#_ENREF_2). Samples were examined at a magnification of 15,000 with a JEOL JEM-1230 transmission electron microscope. For comparison of mitochondrial fission, EM micrographs of thin sections were evaluated. The size of individual mitochondrion was measured by using Image-Pro Plus software. Approximately 1200-1500 mitochondria were measured to determine the percentages of mitochondria with various sizes. In I/R treated heart tissues, mitochondria disintegrated into numerous small round fragments of varying size, the number of small mitochondrion was increasing. Thus, we determined the mitochondria with size less than 0.6 mm^2^ as fission mitochondria. Data represent mean ± SEM of at least three independent experiments.

**References**

1. Tan WQ, Wang K, Lv DY, Li PF. Foxo3a inhibits cardiomyocyte hypertrophy through transactivating catalase. *The Journal of biological chemistry* 2008, **283**(44)**:** 29730-29739.

2. Wang JX, Jiao JQ, Li Q, Long B, Wang K, Liu JP*, et al.* miR-499 regulates mitochondrial dynamics by targeting calcineurin and dynamin-related protein-1. *Nat Med* 2011, **17**(1)**:** 71-78.

3. Li PF, Li J, Muller EC, Otto A, Dietz R, von Harsdorf R. Phosphorylation by protein kinase CK2: a signaling switch for the caspase-inhibiting protein ARC. *Mol Cell* 2002, **10**(2)**:** 247-258.

4. Wang K, Zhou LY, Wang JX, Wang Y, Sun T, Zhao B*, et al.* E2F1-dependent miR-421 regulates mitochondrial fragmentation and myocardial infarction by targeting Pink1. *Nature communications* 2015, **6:** 7619.

5. Frank S, Gaume B, Bergmann-Leitner ES, Leitner WW, Robert EG, Catez F*, et al.* The role of dynamin-related protein 1, a mediator of mitochondrial fission, in apoptosis. *Dev Cell* 2001, **1**(4)**:** 515-525.

6. Wang JX, Li Q, Li PF. Apoptosis repressor with caspase recruitment domain contributes to the chemotherapy resistance by abolishing mitochondrial fission mediated by dynamin-related protein-1. *Cancer Res* 2009, **69:** 492-500.

**Supplementary Figure Legends**

**Supplementary Figure 1. Knockdown of Mtfr1 attenuates mitochondrial fission and apoptosis.** (**A** and **B**) Cardiomyocytes were infected with adenoviruses harboring Mtfr1 siRNA or its sc form and then treated with A/R. The cells with fragmented mitochondria were counted (A). TUNEL-positive cells were calculated (B). *P<0.05 vs A/R alone. (**C** and **D**) Echocardiographic analysis. After intracoronary delivery of adenoviruses harboring Mtfr1 siRNA or the scrambled form, the mice were subjected to I/R injury. Transthoracic echocardiographic analysis was performed. Systolic left ventricular internal diameters (LVIDs). Diastolic left ventricular internal diameters (LVIDd). *P<0.05 vs I/R alone.

**Supplementary Figure 2. MiR-324-5p inhibits apoptosis in cardiomyocytes.** (**A** and **B**) miR-324-5p has no effect on the mRNA levels of Mtfr1. Cardiomyocytes were transfected with miR-324-5p mimic (A) or miR-324-5p antagomir (B). Mtfr1 levels were detected by qRT-PCR. (**C**) miR-324-5p mimic induces the increased miR-324-5p levels. Cardiomyocytes were transfected with miR-324-5p mimic and its negative control, and then were treated with A/R. MiR-324-5p levels were detected by qRT-PCR. *P<0.05 vs control. (**D**) Cardiomyocytes were transfected with miR-324-5p mimic or NC and were treated with A/R. Apoptosis was assayed by caspase-3 activity. *P<0.05 vs A/R alone.

**Supplementary Figure 3. MiR-324-5p decreases myocardial infarct sizes in response to I/R.** (**A**) Adult C57BL/6 mice received miR-324-5p mimic or mimic-NC for 3 days. They were then subjected to I/R. Infarct sizes were calculated. *P<0.05 vs I/R alone. (**B-D**) Echocardiographic analysis. Mice were treated as described for (A), and echocardiography was employed to test heart function. Diastolic left ventricular internal diameters (LVIDd, B). Systolic left ventricular internal diameters (LVIDs, C). Fractional shortening (FS, D) were calculated. *P<0.05 vs I/R alone.

**Supplementary Figure 4. NFAT4 suppresses miR-324-5p expression.** (**A** and **B**) Cardiomyocytes were infected with adenoviral β-gal or NFAT4. NFAT4 expression was analyzed by immunoblot (A). MiR-324-5p levels were analyzed by qRT-PCR (B). *p<0.05 vs control. (**C** and **D**) Cardiomyocytes were infected with adenoviral NFAT4-siRNA or NFAT4-sc. NFAT4 expression was analyzed by immunoblot (C). MiR-324-5p levels were analyzed by qRT-PCR (D). *p<0.05 vs control.

**Supplementary Figure 5. NFAT4 regulates Mtfr1 expression.** (**A**) Cardiomyocytes were infected with adenoviral β-gal or NFAT4. Mtfr1 expression was analyzed by immunoblot. (**B**) Cardiomyocytes were infected with adenoviral NFAT4-siRNA or NFAT4-sc. Mtfr1 expression was analyzed by immunoblot.

**Supplementary Figure 6.** **Knockdown of NFAT4 attenuates mitochondrial fission and apoptosis**. (**A**) Cardiomyocytes were treated with A/R at indicated time. NFAT4 levels were detected by immunoblot. (**B**) Cardiomyocytes were infected with adenoviral NFAT4-siRNA or NFAT4-sc, and then exposed to A/R. NFAT4 levels were detected by immunoblot. (**C** and **D**) Cardiomyocytes were infected with adenoviruses harboring NFAT4-siRNA or its sc form and then treated with A/R. The cells with fragmented mitochondria were counted (C). TUNEL-positive cells were calculated (D). *P<0.05 vs A/R alone.

**Supplementary Figure 7.** **Knockdown of NFAT4 induced the increase of miR-324-5p levels**. (**A** and **B**) After intracoronary delivery of adenoviruses harboring NFAT4 siRNA or the scrambled form, the mice were subjected to I/R injury. NFAT4 levels were analyzed by immunblot (A). miR-324-5p levels were analyzed by qRT-PCR (B). *P<0.05 vs I/R alone.
