# Supplementary figures and images for: NFAT4-dependent miR-324-5p regulates mitochondrial morphology and cardiomyocyte cell death by targeting Mtfr1
Source: Cell Death Dis. 2015 Dec 3;6(12):e2007–. doi: 10.1038/cddis.2015.348 (PMC4720883; doi:10.1038/cddis.2015.348)

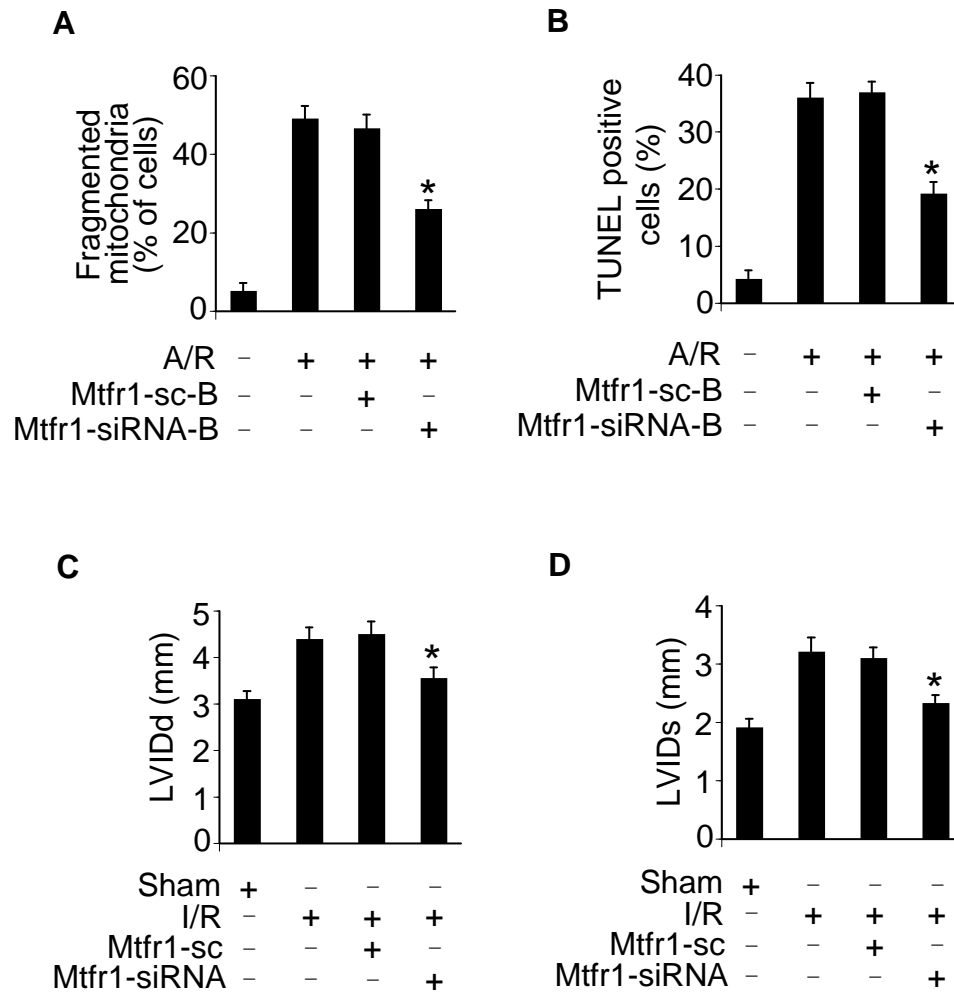

Supplementary Figure 2

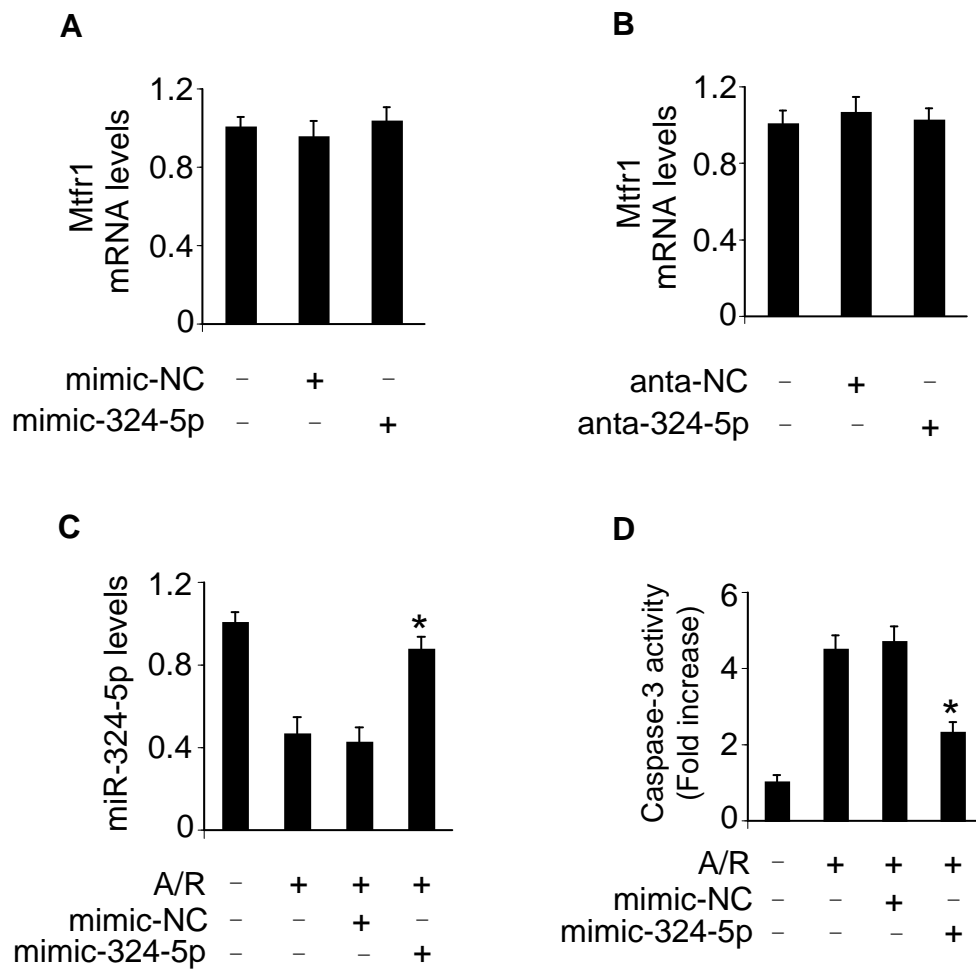

Supplementary Figure 3

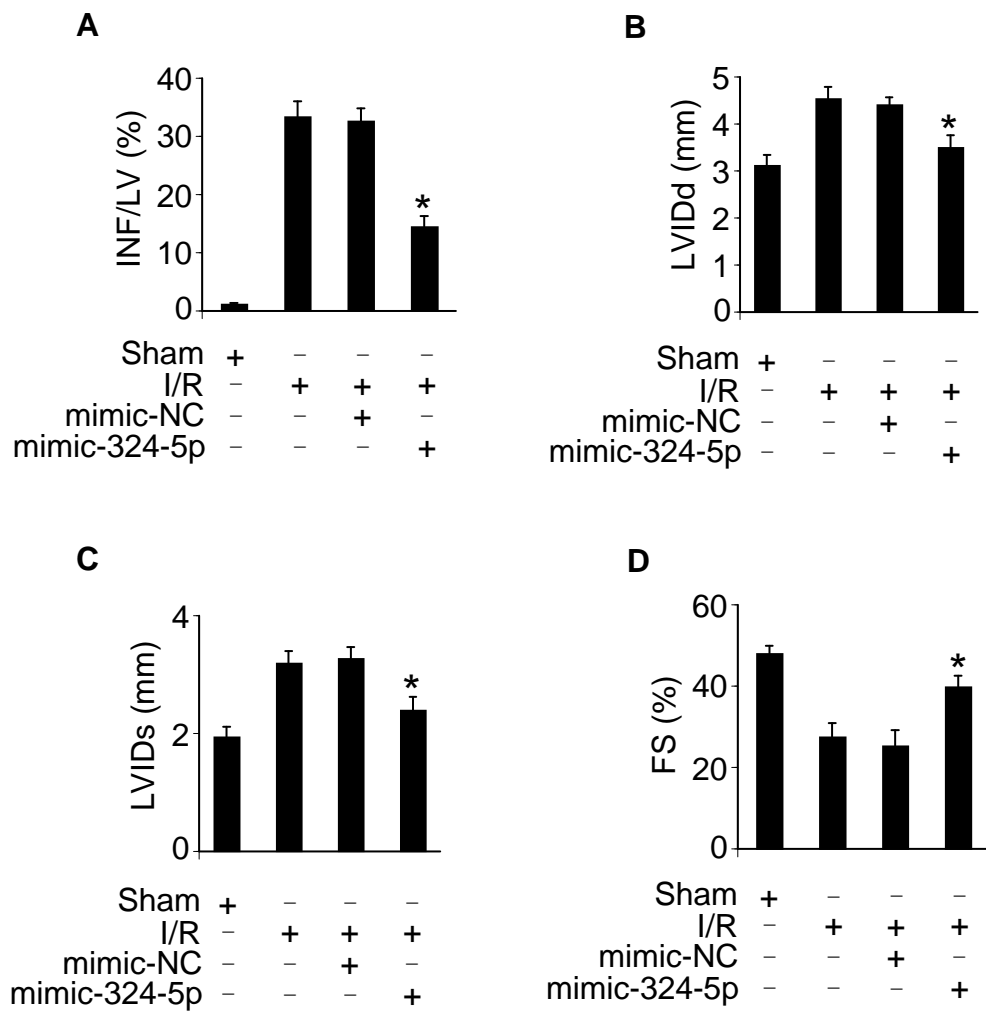

Supplementary Figure 4

**A**

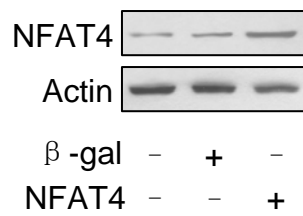

**B**

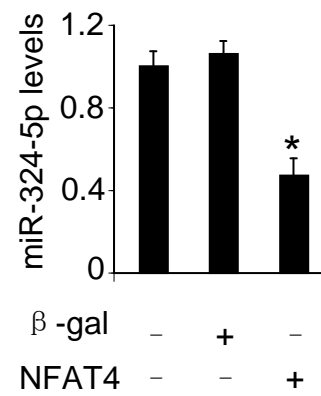

**C**

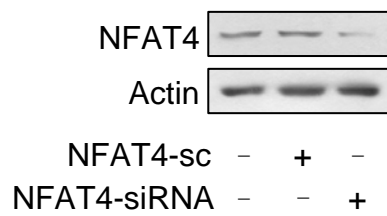

**D**

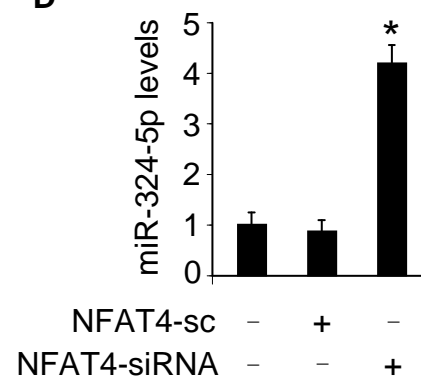

**A**

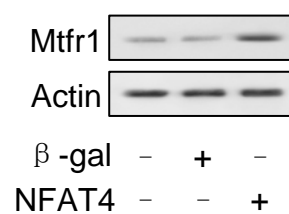

**B**

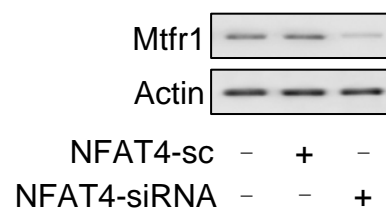

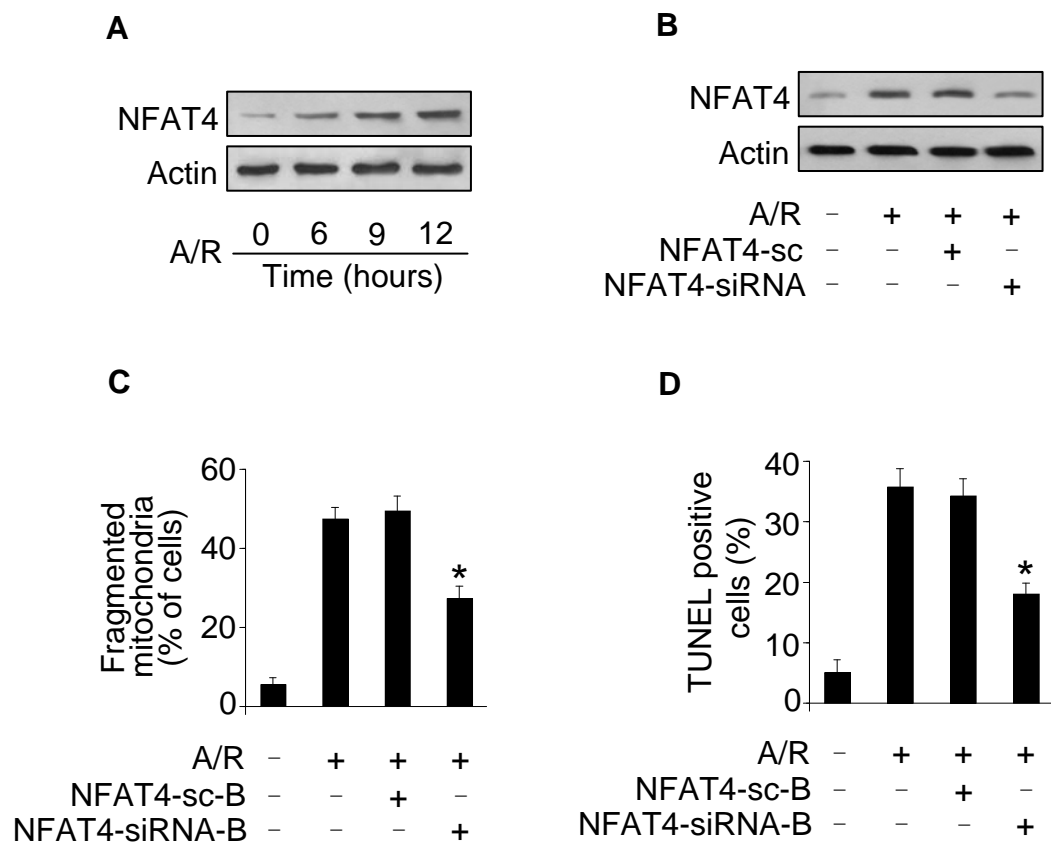

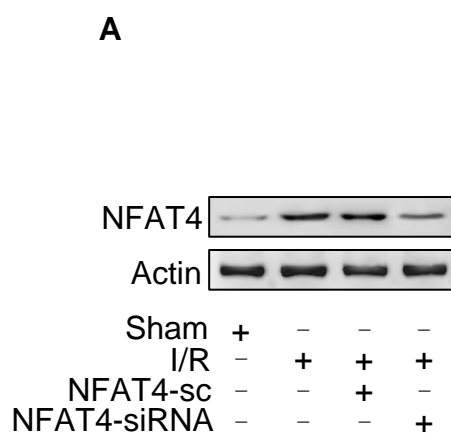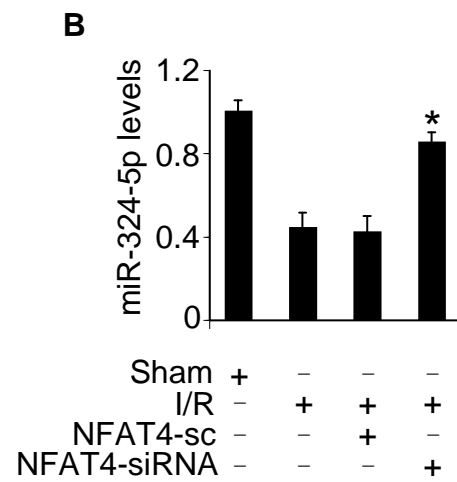

Supplement: Supplementary Figures [file cddis2015348x2.pdf]
